# First Speleomycological Study on the Occurrence of Psychrophilic and Psychrotolerant Aeromycota in the Brestovská Cave (Western Tatras Mts., Slovakia) and First Reports for Some Species at Underground Sites

**DOI:** 10.3390/biology10060497

**Published:** 2021-06-02

**Authors:** Rafał Ogórek, Mateusz Speruda, Justyna Borzęcka, Agata Piecuch, Magdalena Cal

**Affiliations:** Department of Mycology and Genetics, University of Wrocław, Przybyszewskiego Street 63-77, 51-148 Wrocław, Poland; mateusz.speruda@uwr.edu.pl (M.S.); justyna.borzecka@uwr.edu.pl (J.B.); agata.piecuch@uwr.edu.pl (A.P.); magdalena.cal@uwr.edu.pl (M.C.)

**Keywords:** Brestovská Cave, aeromycota, air quality, psychrophilic and psychrotrophic species

## Abstract

**Simple Summary:**

Fungi in underground environments usually occur as spores suspended in the air, because these ecosystems are characterized by harsh living conditions, e.g., constant low temperature, and a limited or complete lack of organic matter and light. Fungi may be potentially hazardous to mammals. Therefore, the goal of our research was the first report of aeromycota in the Brestovská Cave. The mycological quality of the air in the cave does not pose a risk to healthy tourists, but some fungal species may be potentially dangerous to people with a weakened immune system. A total of 18 cold-adapted fungal species were isolated during the study, and all of them were present inside the cave, but only seven were found outdoors. The cosmopolitan species dominated in the external air samples. Our research has allowed for the first detection of fungal species in the air inside the underground sites as well as species that have never been detected in any component of the underground ecosystems. There are many possible reasons explaining the detection of those species, but global warming is the most likely.

**Abstract:**

Most underground ecosystems are heterotrophic, fungi in these objects are dispersed in the air in the form of spores, and they may be potentially hazardous to mammals. Research in underground sites has focused on mesophilic airborne fungi and only a few concerned cold-adapted species. Therefore, the goal of our research was the first report of psychrophilic and psychrotolerant aeromycota in the Brestovská Cave using culture-based techniques with genetic and phenotypic identification. Plates with PDA medium containing sampled biological material were incubated at 8 ± 0.5 °C. The density of mycobiota inside the cave ranged from 37.4 to 71 CFU 1 m^−3^ of air and 63.3 CFU 1 m^−3^ of air outside the cave. Thus, the level of fungal spores did not exceed the standards for the mycological quality of the air. A total of 18 species were isolated during the study, and some species may be potentially dangerous to people with weakened immune system. All fungal species were present inside the cave and only seven of them were outside. *Cladosporium cladosporioides* dominated in the external air samples and *Mortierella parvispora* was cultured most frequently from internal air samples. To our knowledge, this is the first discovery of the fungal species such as *Coniothyrium pyrinum*, *Cystobasidium laryngis*, *Filobasidium wieringae*, *Leucosporidium drummii*, *M. parvispora*, *Mrakia blollopis*, *Nakazawaea holstii*, and *Vishniacozyma victoriae* in the air inside the underground sites. Moreover, *C.* *pyrinum*, *C. laryngis*, *L.* *drummii*, *M. blollopis*, and *N.* *holstii* have never been detected in any component of the underground ecosystems. There are possible reasons explaining the detection of those species, but global warming is the most likely.

## 1. Introduction

Underground ecosystems, both natural and anthropogenic, are characterized by harsh living conditions, which only few microorganisms can cope with, mainly bacteria and microscopic fungi [1,2]. Their species diversity depends strictly on the type of the studied object (natural, anthropogenic), geographic location, or season of the year, which has an impact on the environmental conditions in its individual zones [2,3]. Generally, the deeper from the entrance to the underground complex, conditions become more demanding and microorganisms need to adapt to these conditions. They must survive in conditions of constant low temperature and high humidity, limited or complete lack of light, limited or even no air exchange with the external environment and increasing CO_2_ content [2,4,5,6,7].

Fungal spores can constitute up to 70% of all indoor air bioaerosol pollutants and *Alternaria*, *Aspergillus*, *Cladosporium*, *Fusarium*, and *Penicillium* are the most common genera of fungi isolated from indoor air in buildings and underground ecosystems [8,9]. The presence of these fungi in the air may lead to a deterioration of its mycological quality inside buildings and underground sites, which adversely affects the health of people visiting such facilities, e.g., it can lead to allergic reactions [10,11]. In order to emphasize the seriousness of the poor indoor air quality problem, the World Health Organization introduced the “sick building syndrome” (SBS) term in 1983—where people develop symptoms or chronic infections from being in a building [12]. Fungi and their secondary metabolites (i.e., mycotoxins) play a major role in SBS [13,14]. However, it should be noted that the air is mainly a transmission environment for fungi, so ultimately, with the participation of gravity, they fall, for example, on the ground on rock surfaces or even on organisms living/residing in them, which may lead to the slow biodegradation of rocks or the infection of animals [15,16]. It is worth mentioning that for most underground ecosystems, insects and bats are the actual vectors of new fungi and bacteria, and not air [11,17,18,19]. This shows how extremely important for the underground ecosystem is the development of mycobiota—the dominant community in the described environment [4,6,20]. 

Understanding the species diversity within the underground ecosystem and its role in geological processes or the interaction between a living organism and a fungus are among the most rapidly developing research trends related to biospeleology [15,21,22,23]. Another, less common direction focuses on the characteristics of psychrophilic and psychrotolerant fungi in underground facilities, which are likely to predominate in such ecosystems [7,24], especially psychrophilic species, with an optimal growth temperature below 15 °C and being characterized by numerous structural and functional changes within the cell [25,26]. Consequently, they are capable of producing, e.g., pigments, proteins, or other organic substances (such as sugars) serving as cryoprotectants. Some of the structures characteristic for psychrophiles, i.e., enzymes with a low activation temperature or INPs (ice-nucleating proteins), have found application in molecular studies and in some industries (such as the food industry in ice cream production) [27,28].

The threat to species diversity and the presence of psychrophilic species in underground ecosystems seems to be due to global warming and the development of tourism [20,29]. The interior of many underground complexes has been adapted for visitors. Such anthropological changes may significantly affect the microclimate present there as well as the composition of micro- and mycobiota [2,6,20]. An example of such anthropogenic modifications may be the introduction of additional lighting to the underground, which may contribute to the temperature rise in a given cave (or a single zone), causing a decrease in the number of some species and an increase in the number of others, which are often pathogenic [2,20,30]. Additionally, some species may be introduced into the ecosystem by tourists from the external environment [20].

The main goal of this research was to assess the psychrophilic and psychrotrophic culturable aeromycota in the Brestovská Cave, which is open to tourists, by determining the number and species composition of fungi in the outdoor and indoor air of this cave. Additionally, we wanted to check (1) whether the mycological quality of air within the investigated cave poses a risk to people health, and (2) whether the isolated species may be potentially allergenic and pathogenic to humans.

## 2. Materials and Methods

### 2.1. Study Area

The Brestovská Cave (Slovak: Brestovská jaskyňa) is located near the Zuberec village in the Tatra National Park in Slovakia at the foot of the western Tatra Mountains [31]. The cave entrance is located at an altitude of 867 m above sea level. It is the largest and only open cave in Orava. The total length of the cave reaches 1890 m, of which 217 m is available to tourists. It represents the underground part of a large hydrologic system formed on the contact of karst and non-karst rocks with the river running through the cave. The inside temperature corresponds to the annual average surface temperature, approximately between 4 and 6 °C [32].

### 2.2. Air Sample Collection 

**Figure 1:**
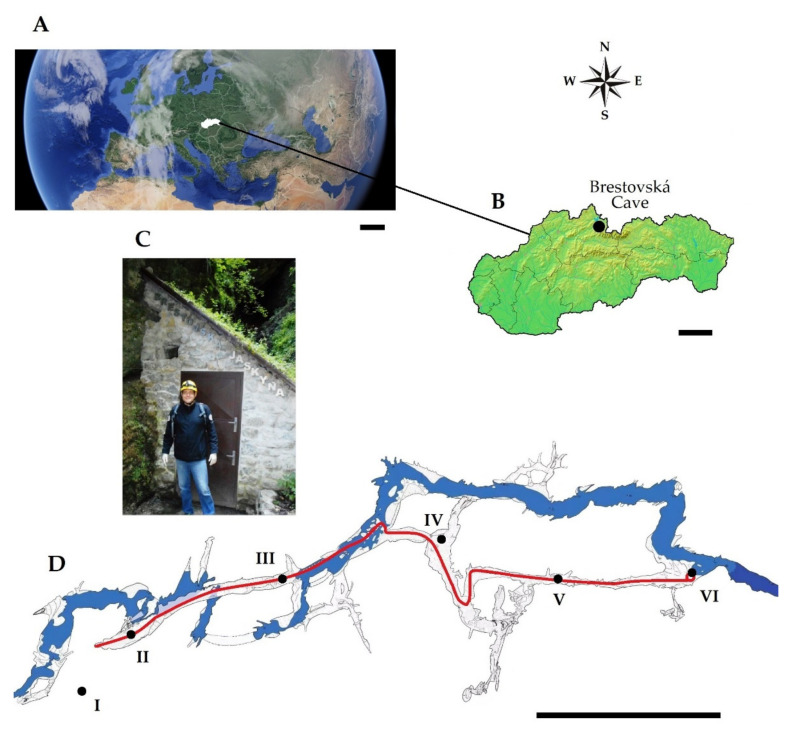
Geographic location of Slovakia (**A**) and the Brestovská Cave (**B**). Entry and exit from the cave (**C**) and underground corridors with marked sampling locations (**D**) on the tourist route (red line): location I outside the cave, and locations II to VI inside the cave. Scale bars: A = 500 km, B = 50 km, C = 50 m.

Field studies were conducted on 24 August 2017. Air samples were collected using a collision method with an AIR IDEAL^®^ 3P^®^ (bioMérieux, Lyon, France) microbial air sampler and PDA medium (Potato Dextrose Agar, BioMaxima, Lublin, Poland) in Petri dishes (⌀ 90 mm). A total of 6 sites were sampled, of which 5 were inside the cave on the route open to the public (sites II to V) and 1 outside (site I)—as shown in Figure 1. The microbial air sampler was positioned at a distance of 1.5 m from above the level of the floor and it was programmed for air sample volumes of 50 L and 100 L. The measurements in a given sampling site were performed in triplicates for each volume.

### 2.3. Isolation of Aeromycota from Samples

Petri dishes with biological material were incubated at 8 ± 0.5 °C for 7 to 42 days in darkness. After incubation, fungal colonies on the plates were counted, and the fungi concentrations were expressed as CFU per cubic meter. Then, the colonies of fungi emerging on the Petri dishes were subcultured on plates with fresh medium and incubated in the dark at 8 ± 0.5 °C for 14 to 28 days. After incubation, fungi were purified by the single spore method and were subcultured on PDA slants for morphological and molecular identification.

### 2.4. Fungal Identification

Firstly, the obtained fungi were identified using classic phenotypic methods according to available monographs and articles [33,34,35,36,37,38,39,40,41,42,43,44,45,46]. In the next step, a molecular analysis of the obtained fungal cultures was performed. For this purpose, DNA was extracted from a 28-day-old culture on PDA using Bead-Beat Micro AX Gravity (A&A Biotechnology, Gdańsk, Poland) according to the manufacturer’s instructions. Fungal rDNA was amplified using two fungal-specific PCR primers: ITS1 (5′-TCCGTAGGTGAACCTGCGG-3′) and ITS4 (5′-TCCTCCGCTTATTGATATGC-3′) [47]. PCR was performed in a T100 Thermal Cycler for 35 cycles (Bio-Rad, Berkeley, CA, USA) according to Ogórek et al. [48]: after initial denaturation for 5 min at 94 °C, each cycle comprised 30 s denaturation at 94 °C, 30 s annealing at 55 °C, 45 s extension at 72 °C with a final extension for 7 min at 72 °C at the end of 35 cycles. The fungal internal transcribed spacer regions were verified by electrophoretic separation on a 1.2% agarose gel, subsequently purified using Clean-Up Kit (A&A Biotechnology, Gdańsk, Poland), and sequenced at Macrogen Europe (The Netherlands, http://dna.macrogen.com/eng/, accessed on 12 May 2021).

### 2.5. Data Analyses

Raw sequence readings were analyzed using the BioEdit Sequence Alignment Editor (http://www.mbio.ncsu.edu/bioedit/bioedit.html, accessed on 12 May 2021). Then, the obtained sequences were compared with those deposited in the GenBank of the National Center for Biotechnology Information (NCBI, Bethesda, Rockville, MD, USA) using the BLAST algorithm (http://www.ncbi.nlm.nih.gov/, accessed on 12 May 2021), and submitted into this database, accessed on 18 September 2020 (Table A1 in Appendix A). 

The obtained data from the number of airborne fungal colonies were analyzed using Statistica 13.0 package (StatSoft Polska Sp. z o.o., Kraków, Poland). For this purpose, one-way analysis of variance (ANOVA) was applied and means were compared using the Tukey HSD (honest significant difference) test at α ≤ 0.05. 

To determine the species diversity of airborne fungi at specific research sites, the Shannon diversity index (H) was used and calculated from the following equation: H = −Σ P_i_(lnP_i_), where Pi stands for the proportion of each species in the sample [49,50]. 

The Pearson correlation coefficient (r) was used to determine the relationship between the Shannon diversity index and the concentration of airborne fungal propagules.

## 3. Results

Speleomycological investigations of airborne fungi in the Brestovská Cave were carried out at one outdoor and five indoor sites using the collision method and the culturable procedure (PDA medium, incubation at 8 ± 0.5 °C) (Figure 1). The phenotypic analysis of the obtained fungal cultures allowed for their identification to 18 species. Molecular studies confirmed the results of phenotypic analyses that all 18 isolates belonged to different species of *Ascomycota* (61.1% isolates), *Basidiomycota* (27.8% isolates), and *Mucoromycota* (11.1% isolates). The cultured species belonged to filamentous fungi (*Chrysosporium merdarium*, *Cladosporium cladosporioides*, *Coniothyrium pyrinum*, *Epicoccum nigrum*, *Mortierella parvispora*, *Mucor hiemalis*, *Oidiodendron truncatum*, *Penicillium brevicompactum*, *P. chrysogenum*, *P. expansum*, *Pseudogymnoascus pannorum*, *Trichoderma viride*), basidiomycetous yeast (*Filobasidium wieringae*, *Leucosporidium drummii*, *Mrakia blollopis*, *Vishniacozyma victoriae*), ascomycetous yeast (*Nakazawaea holstii*) and basidiomycetous yeast-like fungi (*Cystobasidium laryngis*). All the fungal ITS rDNA nucleotide sequences obtained in the study were submitted to GenBank under the accession numbers from MW019461 to MW019478. Based on a BLAST analysis, the E values equaled zero, and the identity range and the percentage of query cover were from 96.40–100% and 100%, respectively (Table A1).

**Figure 2:**
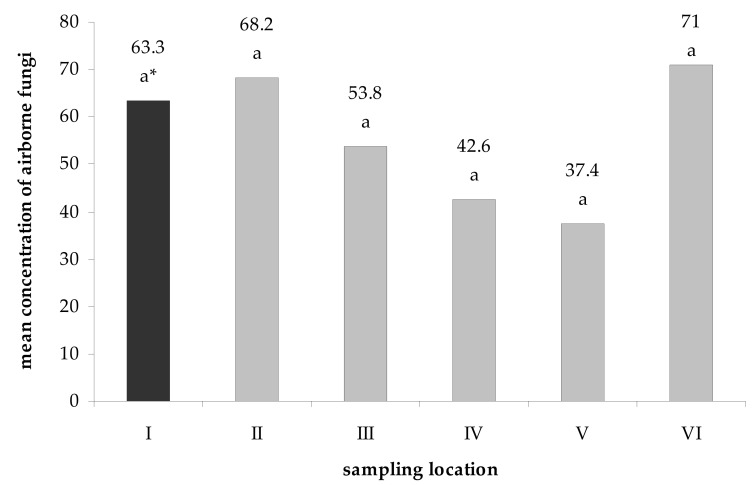
Mean concentration of airborne fungi (CFU per 1 m^3^) cultured from the internal (I) and external air samples (II–VI) of the Brestovská Cave. * For each location, the number of fungal spores followed by the same letter are not statistically different, and others are (Tukey HSD test, α ≤ 0.05). Letters indicate the differences between fungal species in a given location.

The density of mycobiota in the Brestovská Cave ranged from 37.4 to 71 CFU · 1 m^−3^ of air inside the underground facility and 63.3 CFU · 1 m^−3^ of outdoors air. However, no statistically significant differences (the Tukey HSD test at á ≤ 0.05) were found between the concentration of fungal spores at particular study locations (Figure 2).

**Figure 3:**
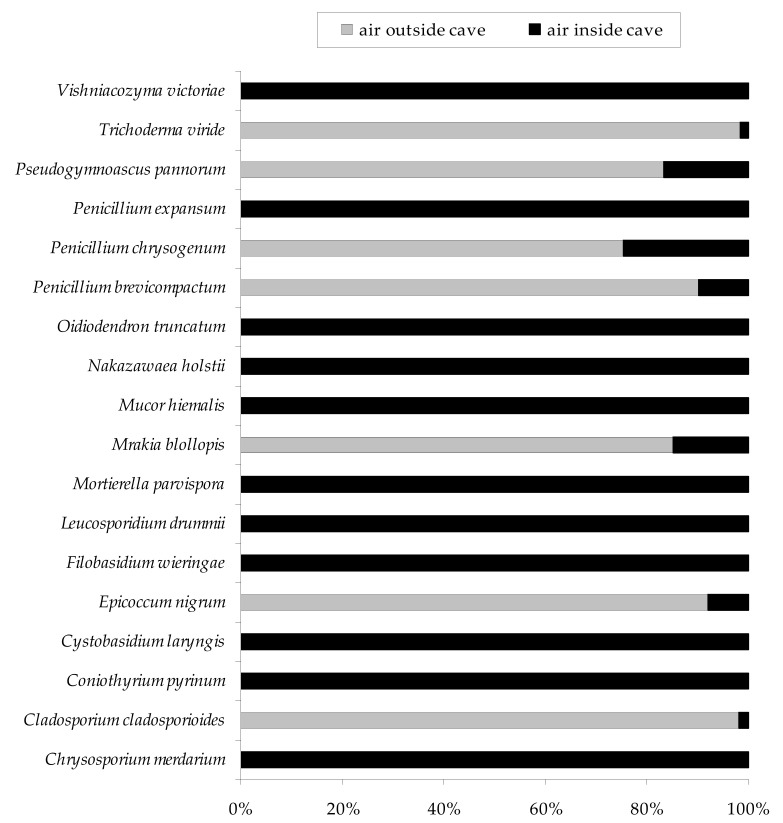
Percentage of each fungal species in the internal and external air samples of the Brestovská Cave.

All 18 fungal species obtained in this study were found inside the Brestovská Cave, and *C. merdarium*, *C. pyrinum*, *C. laryngis*, *F. wieringae*, *L. drummii*, *M. parvispora*, *M. hiemalis*, *N. holstii*, *O. truncatum*, *P. expansum*, and *V. victoriae* were isolated only from indoor air samples (they were not present outdoors). On the other hand, only 7 out of 18 species were detected in outdoor air samples, and these were *C. cladosporioides*, *E. nigrum*, *M. blollopis*, *P. brevicompactum*, *P. chrysogenum*, *P. pannorum*, and *T. viride*. The spores of these species were present in large excess in the air outside the Brestovská Cave compared to its interior, which constituted from 75.5 to 98.3% (Figure 3).

**Figure 4:**
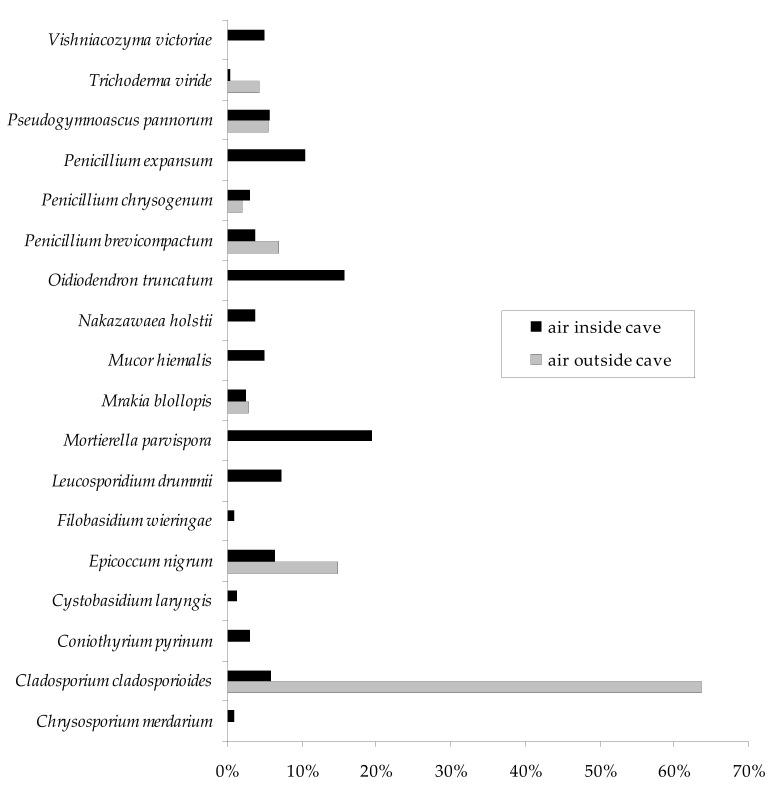
Percentage of each fungal species contributing to the totals from the internal and external air samples of the Brestovská Cave.

The spores of *C. cladosporioides* dominated in the air outside the Brestovská Cave and accounted for 63.7% of all fungal spores caught outside the subterranean site (p *_C._*_*cladosporioides*, *E. nigrum*_ = 0.000178) (Table 1, Figure 4). Overall, *M. parvispora* was cultured most often from internal air samples, and its spores accounted for 19.4% of all isolated fungi inside the cave (Figure 4). However, this species did not dominate in all underground research sites (Table 1). The presence of its spores was detected most often in the study site no. II (p *_M. parvispora_*_, *E. nigrum*_ = 0.000179), and in the study site no. III, it was isolated at a similar level to *C. cladosporioides* and *L. drummii* (p *_L. drummii_*_, *P. pannorum*_ = 0.000763). In turn, *C. pyrinum*, *E. nigrum*, *M. hiemalis*, *O. truncatum*, *P. brevicompactum*, *P. expansum*, and *P. pannorum* were detected most frequently at the same statistical level in the study site no. IV (p *_O. truncatum_*_, *P. chrysogenum*_ = 0.006640), and *M. hiemalis*, *O. truncatum*, *P. chrysogenum*, *P. expansum*, and *P. pannorum* in the study site no. V (p *_O. truncatum_*_, *M. blollopis*_ = 0.000869). *Oidiodendron truncatum* and *P. expansum* were also dominant in the study site no. VI (p *_O._*_*truncatum*, *V. victoriaes*_ = 0.000450) (Table 1).

**Figure 5:**
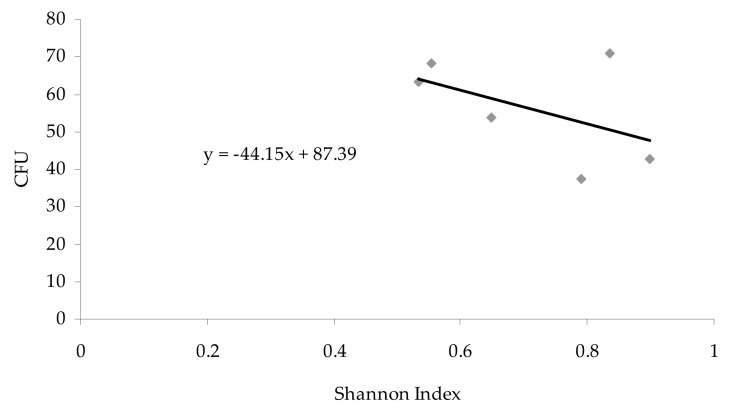
Relationship between the number of airborne fungal spores (CFU 1 m^−3^ of air) and Shannon index in the Brestovská Cave (the Pearson correlation coefficient: *p* < 0.05; r = −0.49).

The studied sites differed from each other in the diversity of fungal species, which is illustrated by the obtained Shannon index. The species diversity of aeromycota inside the cave (from 0.5542 to 0.8977) was greater than outside (0.5329), and the highest value of Shannon index was noted in the study site no. IV (Table 1). However, there was no positive correlation (*p* < 0.05; r = −0.49) between the Shannon diversity index and the concentrations of airborne fungal propagules (Figure 5).

## 4. Discussion

The effectiveness of the culture-based analysis of fungal species depends largely on the incubation temperature and the type of culture medium [51,52]. The fungal response to temperature is quite diversified. Generally, most fungi grow well at a room temperature ranging from 20 to 25 °C, but some species, such as psychrophilic and psychrotolerant fungi, prefer cooler temperatures and they most likely predominate in underground ecosystems [7,24,25,26]. The purpose of this work was to assess the mycobiota not only transferred to the Brestovská Cave from the external environment but also to analyze species that have the potential to live there. Therefore, the incubation temperature used in this study (8 °C) was directed towards psychrophilic and psychrotolerant fungi. Although Sabouraud agar medium is most suitable for the large spectrum isolation of fungi the environment [53], we used PDA medium since it demonstrates comparable efficacy as Sabouraud agar [54], and it is now one of the most commonly used media in speleomycological research [7,9,11,16,17]. This medium is composed of dehydrated potato infusion and dextrose that encourage luxuriant fungal growth and pigment production in some dermatophytes. Agar is added as the solidifying agent. Additionally, it can be supplemented with acid or antibiotics to inhibit bacterial growth [54,55].

Generally, underground sites are considered to be heterotrophic ecosystems, with few exceptions, e.g., the Movile Cave “Peștera Movile” in Romania being a sulfur-based chemolithoautotrophic ecosystem [56]. Fungi in underground environments usually occur as spores and other propagation structures suspended in the air and are transferred there with air currents from the external environment [11,15,16], which is a very important issue, since some of the air biological components can be the cause of human illness, as already mentioned by Hippocrates in the *Corpus Hippocraticum* manuscript [57]. The presence of fungi and their secondary metabolites can significantly reduce the quality of air; however, it depends on the fungal species and the number of spores [58,59]. 

The concentration of psychrophilic and psychrotolerant aeromycota in the Brestovská Cave ranged from 37.4 to 71 CFU per 1 m^3^ of indoor air and 63.3 CFU per 1 m^3^ of outdoor air. Currently, there are no official norms, standards or proposals for mycological air quality in underground facilities; however, there are such documents concerning the indoor air of buildings, but without the specifications regarding fungal preferences for the growth temperature, e.g., the WHO, American Industrial Hygiene Association, the European Confederation Commission, or the Polish norm PN89/Z-04111/03. The concentration of aeromycota should not exceed 500 spores in 1 m^3^ of air according to the most stringent standard among the aforementioned organizations [12,58,59,60]. Thus, the mycological quality of air in the Brestovská Cave does not pose a risk to healthy tourists. Moreover, the concentration of aeromycota detected in our study was at a similar or lower level as in other caves in Slovakia studied during summer with an air sampler and PDA medium [9,17,61,62,63]. 

The values of fungal spore contaminations obtained in our study were lower or similar to those inside other Slovakian caves (Harmanecká Cave, Demänovská ľadová Cave, Demänovská slobody Cave), and much lower than those obtained outside these objects during summer by using PDA medium and an AIR IDEAL^®^ 3P^®^ microbial air sampler. However, the above-mentioned studies applied different incubation temperatures than those used in this work [9,62,63]. In the Demänovská ľadová and Demänovská slobody Caves studies, the samples were incubated at 25 °C, and the outside air contained 755 and 391 CFU per 1 m^3^, respectively [62,63]. In turn, the outside air samples from the Harmanecká Cave were incubated at 15, 20 and 25 °C, and the air contained more than 810 CFU per 1 m^3^ [9]. Probably, the incubation temperature of the samples was the main factor determining the species composition and the number of fungi obtained in those studies. The atmospheric air in summer contains mainly mesophillic species for whose growth, an incubation temperature around 25 °C is optimal [9,62,63].

Various studies have shown that the species diversity of aeromycota is much higher inside underground sites than outside, regardless of the type of object, its function, and inhabiting animals [6,7,11,64]. Our research is in agreement with those observations since all of the 18 isolated fungal species were present inside the Brestovská Cave, and only seven of them were discovered outside, as illustrated by the Shannon diversity index values. Although, it should be noted that there was no positive correlation between the diversity index and the concentration of airborne fungi. 

Our research is also consistent with the reports of Vanderwolf et al. [8], who proved that fungi belonging to the phylum *Ascomycota* dominate in underground sites where they constitute ca. 69% of all cultured fungi. In addition, we isolated fungal species belonging to groups with various morphological forms such as filamentous fungi, ascomycetous and basidiomycetous yeast, and basidiomycetous yeast-like fungi. Nevertheless, filamentous fungi constituted the vast majority, which confirms previous scientific reports [6,7,8,11,64]. 

The incubation temperature of the samples (8 °C) in this research probably also influenced the diversity of fungal species since it is optimal for the growth of psychrophilic fungi [25,26]. Therefore, the diversity of aeromycota inside the Brestovská Cave was greater than outside, and it increased with the elevation of the distance from the entrance to the cave interior. As a consequence, the dominance of cold-adapted fungal species increased with the depth of the cave. On the other hand, the growth of fungal species occurring outside the Brestovská Cave was limited by the temperature of incubation used in our study because during summer, mesophilic fungi dominate in the air [9,62,63].

The spores of *C. cladosporioides* dominated in the external air samples of the Brestovská Cave. Aeromycological investigations of the atmosphere as well as the indoor air of the buildings but also the underground sites show that the fungal propagules of *Cladosporium* are most frequently detected [65,66,67]. Its conidia are one of the most allergenic biological particles in the air, and can be the cause of allergic rhinitis, asthma, or allergic alveolitis. However, our studies showed that the level of *Cladosporium* spores detected in the air of the Brestovská Cave does not constitute a significant allergic risk to people, since a minimum of 2800 *Cladosporium* spores per 1 m^3^ of air is required for the emergence of respiratory allergies in humans [68]. 

In turn, the spores of *M. parvispora* were cultured most frequently from internal air samples of the Brestovská Cave. This species along with others such as *Coniothyrium pyrinum*, *Cystobasidium laryngis*, *Filobasidium wieringae*, *Leucosporidium drummii*, *Mrakia blollopis*, *Nakazawaea holstii*, and *Vishniacozyma victoriae* were discovered for the first time in the air of undergrads sites, according to our knowledge. Moreover, *C. pyrinum*, *C. laryngis*, *L. drummii*, *M. blollopis*, and *N. holstii* have never been detected so far in any site of the underground ecosystems. It is difficult to explain the occurrence of these new species. One of the reasons for their detection may be the accuracy of research methods, but it can also be associated with anthropogenic factors, as well as the global warming, which may be responsible for the rise of the temperature such types of objects [29]. Probably, the increase in temperature in underground sites, as in other ecosystems, contributes to a variation in the inhabiting microbial communities and may lead to the appearance of new species, including those pathogenic for plants and mammals [69,70].

*Filobasidium wieringae* was found in ice caves in Antarctica, but never before from the air of underground sites [71]. Similarly, *M. parvispora* and *V. victoriae* were isolated from places related to underground ecosystems, but never from the air of such objects [27,72]. The former was isolated from wooden timbers and the latter from wood materials as well as from visible fungal mycelia or rhizomorphs of an underground Soudan iron mine in Lake Vermilion Soudan Underground Mine State Park in Tower Minnesota [72]. In turn, *V. victoriae* was isolated from bent-winged bats (*Miniopterus orianae bassanii* and *oceanensis*) in a cave in South Australia, and from the perennial ice block of Scărișoara Ice Cave [27,73]. 

Most of the completely newly isolated species in the underground sites were previously associated with cold environments, soil or plants. Antarctic soil was also inhabited by *C. laryngis* and *M. blollopis* [44,74]. Additionally, *C. laryngis* was isolated from barley grains [75]. *Leucosporidium drummii* was firstly described in Germany as soil isolate; however, it was suggested that the soil is not its primary habitat. Fungi of Leucosporidiales are usually psychrotolerant inhabitants of water and plant material [43]. *Nakazawaea holstii* is another environmental fungus, found in soil, lakes and streams, and it was isolated from olive fruits [76,77]. In turn, *C. pyrinum* was cultured from severe dieback and cankers, which appeared on young and old branches of almond trees [78]. 

Newly isolated fungi from the Brestovská Cave generally does not pose a threat to human health; however, *V. victoriae* was detected in domestic environments and it was suggested to be associated with asthma [79]. On the other hand, our research confirms that the genus *Penicillium* is the most numerous group, in terms of the number of species, isolated from underground ecosystems [7,11]. These fungi are dangerous to humans and other mammals because they secrete and produce numerous conidial spores and mycotoxins, which enter the body with air and food. Moreover, they can settle on various components of the environment and along with other fungi, e.g., *Aspergillus*, are one of the most important biological factors contributing to SBS [80,81,82]. All isolated *Penicillium* species in the study (*P. chrysogenum*, *P. brevicompactum*, and *P. expansum*) are a frequent component of bioaerosol in underground ecosystems [7,8,64,67]. *Penicillium chrysogenum* can only be dangerous to people with a weakened immune system (e.g., in transplant recipients), causing pneumonia [83,84]. *Penicillium brevicompactum*, such as *P. chrysogenum*, in very exceptional situations may cause pneumonia, e.g., in an allogeneic bone marrow transplant recipient or in a young Staffordshire bull terrier [85,86]. Additionally, *P. expansum* is mainly considered as a global postharvest pathogen causing blue mold of pome fruit with a strong potential for the production of patulin, chaetoglobosins, and other secondary metabolites [87,88].

## 5. Conclusions

Our study contributes to gaining new knowledge about psychrophilic and psychrotolerant airborne fungi inhabiting caves in the summer. The concentrations of fungi in the air of the investigated sites were on similar levels and do not pose a health risk for people. Overall, we isolated 18 fungal species, and all of them were present inside the cave, but only seven were found outdoors. There was no positive correlation between the Shannon diversity index and the concentration of aeromycota, and the dominance of cold-adapted fungal species increased with the depth of the cave. The cosmopolitan species such as *Cladosporium cladosporioides* dominated in the external air samples. In turn, little-known *Mortierella parvispora* was cultured most frequently from internal air samples. Our research has allowed for the first detection of fungal species in the air inside the underground sites (*Coniothyrium pyrinum*, *Cystobasidium laryngis*, *Filobasidium wieringae*, *Leucosporidium drummii*, *M. parvispora*, *Mrakia blollopis*, *Nakazawaea holstii*, and *Vishniacozyma victoriae*), as well as new species in any component of the underground ecosystems (*C. pyrinum*, *C. laryngis*, *L. drummii*, *M. blollopis*, and *N. holstii*). There are many reasons explaining the detection of those species, but global warming is the most likely.

## Figures and Tables

**Table 1:** Average number of airborne fungal propagules (CFU m^−3^) in the Brestovská Cave: I—the outdoor air samples, II–VI—the indoor air samples; ND = not detected. * For each location, the number of fungal spores followed by the same letter are not statistically different, and others are (Tukey HSD test, α ≤ 0.05). Letters indicate the differences between fungal species in a given location.

Fungal Species	Sampling Location					
	I	II	III	IV	V	VI
*Chrysosporium merdarium*	ND	ND	ND	2.2 b	ND	ND
*Cladosporium cladosporioides*	40.3 a*	4.1 b	11.9 ab	ND	ND	ND
*Coniothyrium pyrinum*	ND	ND	5.3 b	2.7 ab	ND	ND
*Cystobasidium laryngis*	ND	ND	ND	ND	ND	3.6 c
*Epicoccum nigrum*	9.4 b	10.6 b	ND	3.4 ab	1.4 b	2.0 c
*Filobasidium wieringae*	ND	ND	1.3 b	ND	1.0 b	ND
*Leucosporidium drummii*	ND	ND	19.8 a	ND	ND	ND
*Mortierella parvispora*	ND	39.7 a	13.4 ab	ND	ND	ND
*Mrakia blollopis*	1.8 b	ND	ND	ND	1.5 b	5.3 c
*Mucor hiemalis*	ND	ND	ND	5.3 ab	4.0 ab	4.4 c
*Nakazawaea holstii*	ND	5.2 b	ND	ND	ND	4.9 c
*Oidiodendron truncatum*	ND	ND	ND	8.7 a	11.3 a	23.0 a
*Penicillium brevicompactum*	4.4 b	ND	ND	8.0 ab	ND	2.2 c
*Penicillium chrysogenum*	1.2 b	ND	ND	2.4 b	4.2 ab	1.8 c
*Penicillium expansum*	ND	ND	ND	2.8 ab	8.0 ab	17.7 ab
*Pseudogymnoascus pannorum*	3.5 b	ND	2.1 b	7.1 ab	6.0 ab	ND
*Trichoderma viride*	2.7 b	1.0 b	ND	ND	ND	ND
*Vishniacozyma victoriae*	ND	7.6 b	ND	ND	ND	6.1 bc
Shannon index	0.5329	0.5542	0.6483	0.8977	0.7898	0.8362

## Data Availability

Not applicable.

## Appendix A

**Table A1:** Cultured airborne fungi detected in the Brestovská Cave and results of BLAST (all E values were zero).

Fungi Isolated from Air Samples					Identity with Sequence from GenBank		
Isolate Number	Identified Species	Phylum	GenBank Accession No.	The Sequence Length (bp)	Query Cover, %	Identity, %	Accession
UWR_189	*Chrysosporium merdarium*	*Ascomycota*	MW019461.1	442	100	100.00	MH859164.1
UWR_190	*Cladosporium cladosporioides*	*Ascomycota*	MW019462.1	377	100	99.20	MT635286.1
UWR_191	*Coniothyrium pyrinum*	*Ascomycota*	MW019463.1	412	100	99.51	MH858918.1
UWR_192	*Cystobasidium laryngis*	*Basidiomycota*	MW019464.1	512	100	100.0	MG674823.1
UWR_193	*Epicoccum nigrum*	*Ascomycota*	MW019465.1	439	100	99.77	MT111108.1
UWR_194	*Filobasidium wieringae*	*Basidiomycota*	MW019466.1	528	100	100.00	MN128850.1
UWR_195	*Leucosporidium drummii*	*Basidiomycota*	MW019467.1	510	100	100.00	KU745364.1
UWR_196	*Mortierella parvispora*	*Mucoromycota*	MW019468.1	398	100	96.40	MT380893.1
UWR_197	*Mrakia blollopis*	*Basidiomycota*	MW019469.1	537	100	100.00	MK693027.1
UWR_198	*Mucor hiemalis*	*Mucoromycota*	MW019470.1	514	100	100.00	MN105537.1
UWR_199	*Nakazawaea holstii*	*Ascomycota*	MW019471.1	527	100	100.00	KY104368.1
UWR_200	*Oidiodendron truncatum*	*Ascomycota*	MW019472.1	435	100	99.77	KF835845.1
UWR_201	*Penicillium brevicompactum*	*Ascomycota*	MW019473.1	520	100	100.00	MN902154.1
UWR_202	*Penicillium chrysogenum*	*Ascomycota*	MW019474.1	453	100	100.00	MT594471.1
UWR_203	*Penicillium expansum*	*Ascomycota*	MW019475.1	505	100	100.00	MT558929.1
UWR_204	*Pseudogymnoascus pannorum*	*Ascomycota*	MW019476.1	552	100	99.28	KP411572.1
UWR_205	*Trichoderma viride*	*Ascomycota*	MW019477.1	544	100	100.00	MK290390.1
UWR_206	*Vishniacozyma victoriae*	*Basidiomycota*	MW019478.1	564	100	100.00	LC515132.1
